# Effects of Simulated Gravel on Hydraulic Characteristics of Overland Flow Under Varying Flow Discharges, Slope Gradients and Gravel Coverage Degrees

**DOI:** 10.1038/s41598-019-56223-2

**Published:** 2019-12-24

**Authors:** Xiaona Liu, Dengxing Fan, Xinxiao Yu, Ziqiang Liu, Jiamei Sun

**Affiliations:** 1 Beijing Forestry University, Key Laboratory of Soil and Water Conservation and Desertification Combating, State Forestry Administration, Beijing, 100083 China; 2 Co-Innovation Center for Sustainable Forestry in Southern China, Nanjing Forestry University, Nanjing, 210037 China; 3 Institute of Botany, Chinese Academy of Sciences, Beijing, 100093 China

**Keywords:** Environmental impact, Hydrology

## Abstract

To quantify the hydraulic characteristics of overland flow on gravel-covered slopes, eight flow discharges (*Q*) (8.44–122 L/min), five slope gradients (*J*) (2°–10°) and four gravel coverage degrees (*Cr*) (0–30%) were examined via a laboratory flume. The results showed that (1) gravel changed flow regime. Gravel increased the Reynolds number (*Re*) by 2.94–33.03%. *Re* were less affected by *J* and positively correlated with *Cr* and *Q*. Gravel decreased the Froude number (*Fr*) by 6.83–77.31%. *Fr* was positively correlated with *Q* and *J* and negatively correlated with *Cr*. (2) Gravel delayed the flow velocity (*u*) and increased the flow depth (*h*) and flow resistance (*f*). Gravel reduced *u* by 1.20–58.95%. *u* was positively correlated with *Q* and *J* and negatively correlated with *Cr*. Gravel increased *h* by 0.12–2.41 times. *h* was positively correlated with *Q* and *Cr* and negatively correlated with *J*. Gravel increased *f* by 0.15–18.42 times. *f* were less affected by *J*, positively correlated with *Cr* and negatively correlated with *Q*. (3) The relationships between hydraulic parameters and *Q*, *J* and *Cr* identified good power functions. Hydraulic parameters were mainly affected by *Cr*. These results can guide the ecological construction of soil and water conservation.

## Introduction

Soil erosion consists of the processes of erosion, destruction, separation, transportation and deposition of soil and its parent materials under external forces^1,2^. Serious soil erosion leads to the loss of fertile topsoil, destroys land resources and reduces land productivity, affecting food and ecological security. Sediment and pollutants carried by erosion processes cause siltation and blockages in rivers and lakes and eutrophication of water bodies, and these effects aggravate drought and flood disasters^3–5^. In addition, the soil nutrient content and composition are impacted by erosion transport, which further affects the global biogenic factor cycle^6–8^. Thus, soil erosion is among the major ecological and environmental problems worldwide^9,10^. Soil erosion is affected by various factors, including the rainfall intensity, topography, soil properties, land cover and other factors. Among these factors, land cover, such as vegetation, litter, and gravel, is the most effective measure for preventing soil erosion^11^.

Gravel includes particles of 2 mm in diameter or larger and with horizontal dimensions of less than one pedon. Gravel cover affects the soil erosion process. Rieke-Zapp *et al*.^12^ suggested that gravel decreased surface runoff and sediment accumulation. Cerda^13^ reported that gravel retarded ponding and led to greater steady-state infiltration rates and smaller sediment concentrations under field conditions using simulated rainfall. Von-Bennewitz *et al*.^9^ indicated that gravel cover contributed to delaying the start time of surface runoff, and showed that the amount of surface runoff was directly proportional to the rainfall intensity and inversely proportional to the gravel coverage degree. However, the results may differ with a high gravel coverage degree. Zhou *et al*.^10^ showed that infiltration rates decreased when the gravel content was less than 40% but increased at values greater than 40%. Zavala *et al*.^11^ stated that soil erosion and runoff generation increased when the gravel coverage degree was greater than 60%. The relationship between gravel cover and the soil erosion process had both positive and negative associations.

Gravel affects soil erosion processes in various ways^13,14^. Direct effects include protection against raindrop splash impact or the interception of splashed sediment^15^, while indirect effects are associated with the physical properties of soil (e.g., porosity, bulk density, and organic matter content), physical degradation of surface soil (e.g., surface sealing and compaction), and hydrological processes that affect runoff generation (e.g., infiltration)^16–18^. Furthermore, the characteristics of gravel itself (e.g., gravel coverage, gravel size, and gravel position in the soil) could also lead to different soil erosion outcomes^12^. A reduction of the gravel coverage degree does not prevent the soil surface from sealing and leads to a decreased infiltration rate^19^. Gravel laying on the soil surface decreases runoff generation and increases the infiltration rate^20,21^, whereas gravel embedded in soil increases the runoff rate^22^. Moreover, the gravel particle size may contribute to plugging soil macropores^23^.

Soil erosion and gravel cover are closely related, especially in rocky mountainous areas where vegetation cover is very low^24^. Gravel is often used as a traditional technique on the soil surface. In Anasazi (northern New Mexico), farmers cover gardens located on eroded terraces with gravel to reduce drought and increase crop yields^25^. In Korhogo and Niangoloko, gravel cover leads to a significant reduction in soil loss during decennial storm events on bare and moist soil surfaces^26^. In the semi-arid and high erosion loess area of Northwest China, gravel cover could change the hydrological processes and improve the soil productivity, and it can facilitate vegetation growth compared with areas without cover^27^. In recent years, gravel cover has been combined with plastic film mulch, drip irrigation, rain water harvesting, and rotation to maximize the water use efficiency in both northwestern China and Las Vegas, Nevada, United States^28^.

Although a large number of studies have been performed to determine the influence of gravel cover on the soil erosion process, the mechanism underlying gravel’s control of runoff and sediment loss remains poorly understood. Studies have confirmed that the functionary mechanism of overland flow scouring soil can be elaborated and illustrated via quantitative studies on the hydraulic characteristics of overland flow, such as the flow velocity, water depth, flow regime, resistance coefficient, etc.^29^. Therefore, the influence of gravel cover on the hydraulic characteristics of overland flow must be elucidated to reveal the functionary mechanism of gravel on soil and water conservation and to guide the ecological construction of soil and water conservation.

## Materials and Methods

### Site description

The experiment was conducted in a rainfall simulation hall at the Key Laboratory of Soil and Water Conservation and Desertification Combating, which is located in Jiufeng National Forestry Mountains, Beijing, China. The laboratory belongs to the Beijing Forestry University, is located on a 10–25% slope and has a temperate continental climate with an average annual temperature of 9 °C. The altitude is 140 m, and the average annual precipitation is 600 mm, of which more than 80% falls between June and September. The active growing season extends from April to October. The soil is a shallow (from 0.3 to 1 m with an average of 0.5 m) gravelly loam with a mean 13.9% coarse fraction (>2 mm diameter), and the highest gravel fraction is 30%^30^.

### Experimental conditions and treatments

**Figure 1:**
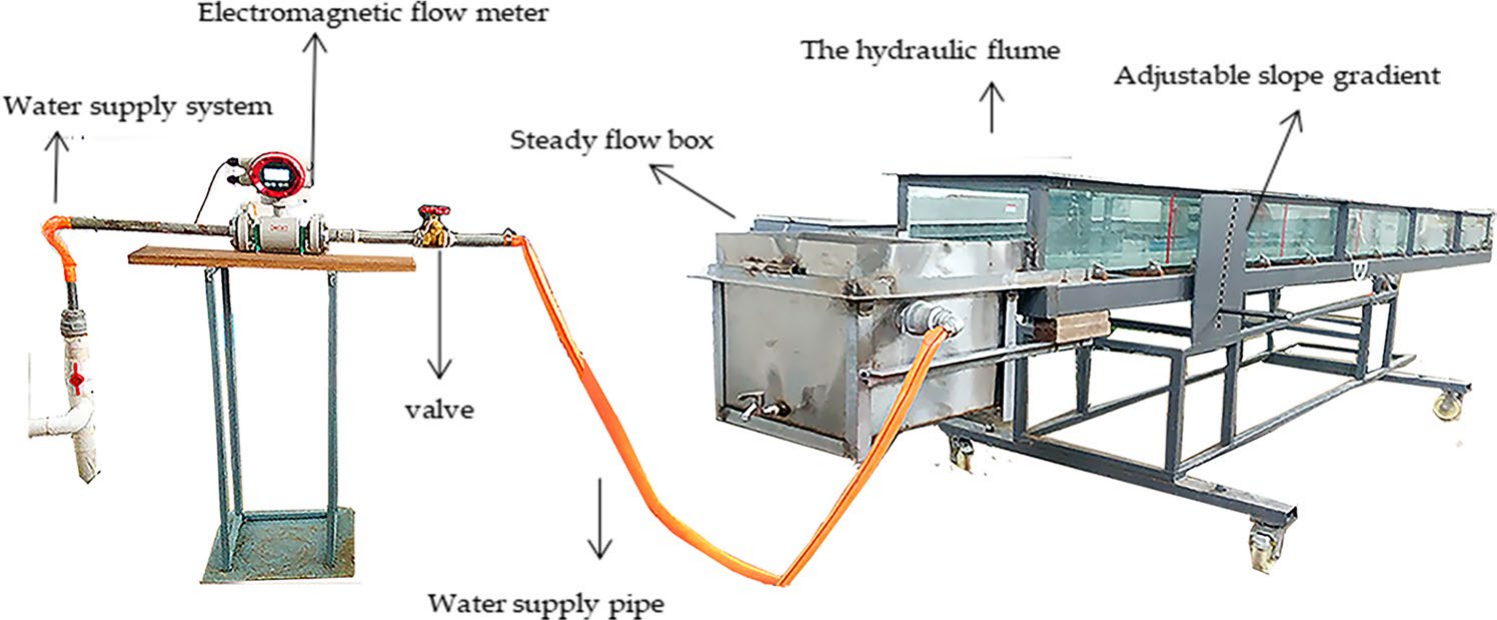
Schematic diagram of the runoff scouring equipment.

All experiments were processed using runoff scouring equipment inside the laboratory in the rainfall simulation hall. Runoff scouring equipment consisted of four main components: a water supply system, a flow discharge control system, flat water facilities, and a hydraulic flume. The flow discharge control system was composed of an electromagnetic flow meter and valve, and it was used to display and control the size of the flow discharge. Flat water facilities included a steady flow box and hump, which allowed the water to flow smoothly into the hydraulic flume. The hydraulic flume was 6 m long, 0.5 m wide, and 0.3 m deep, and it had 12-mm-thick tempered glass on the bottom and could be adjusted to slope gradients from 0 to 15°. The schematic diagram is shown in Fig. 1.

**Figure 2:**
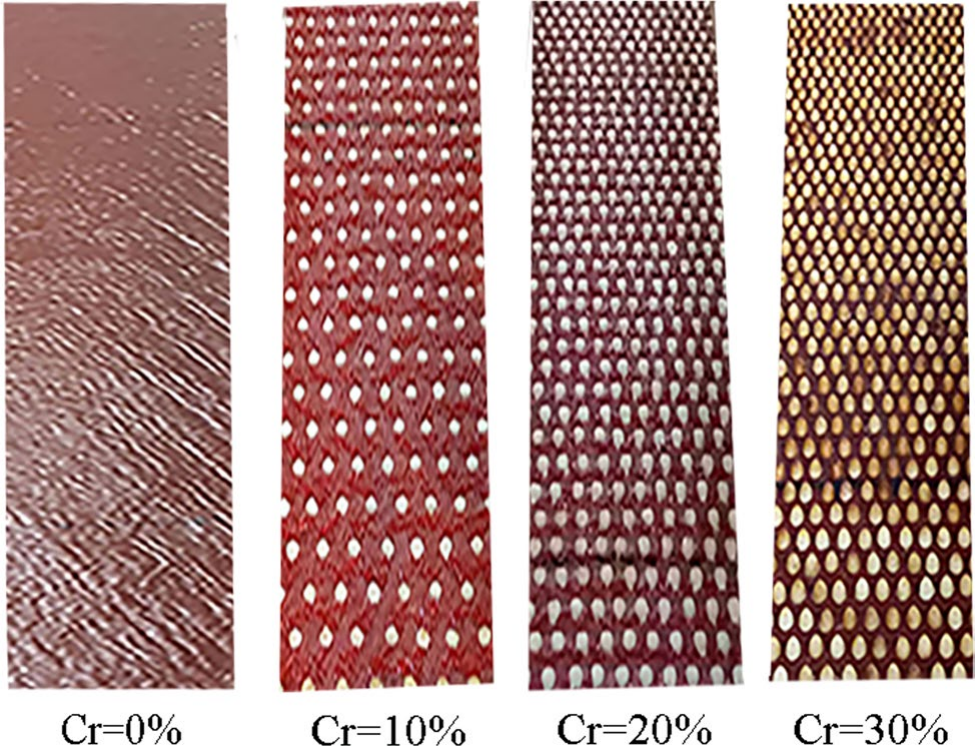
Schematic diagram of the four underlying surfaces.

Four underlying surfaces were used in the hydraulic flume: a bare slope as the control and three gravel-covered slopes (Fig. 2). Sixty mesh sand cloths with a particle size of 0.25 mm were placed on the bottom of the hydraulic flume to simulate the underlying soil surface. Well-defined elements, such as plastic hemispheres, were used in this study to accurately describe the geometric characteristics of natural gravel^31^. Plastic hemispheres with a diameter of 2 cm and a certain roughness were evenly affixed to the sand cloth in a plum blossom arrangement to simulate gravel-covered slopes. Considering the natural gravel content, the maximum gravel coverage degree was designed to be 30% in this experiment. Generally, four levels of gravel coverage (0%, 10%, 20%, and 30%) were implemented.

Considering the range of rainfall intensities and the slope gradient in rocky mountainous areas of North China^30^, five slope gradients (2°, 4°, 6°, 8° and 10°) and eight flow discharges (8.44, 11.26, 22.52, 45.03, 70.36, 84.43, 100 and 122 L/min) were utilized in this experiment. A total of 160 group tests were conducted with a complete combination test.

### Experimental measurements

Prior to each test, the slope gradient and flow discharge were adjusted to the designed value. The flow discharge was controlled by an electromagnetic flow meter, and the valve and volume method was utilized to calibrate the flow discharge at the outlet end of the hydraulic flume. Measurements of flow depth and water temperature were performed after the flow discharge stabilized.

**Figure 3:**
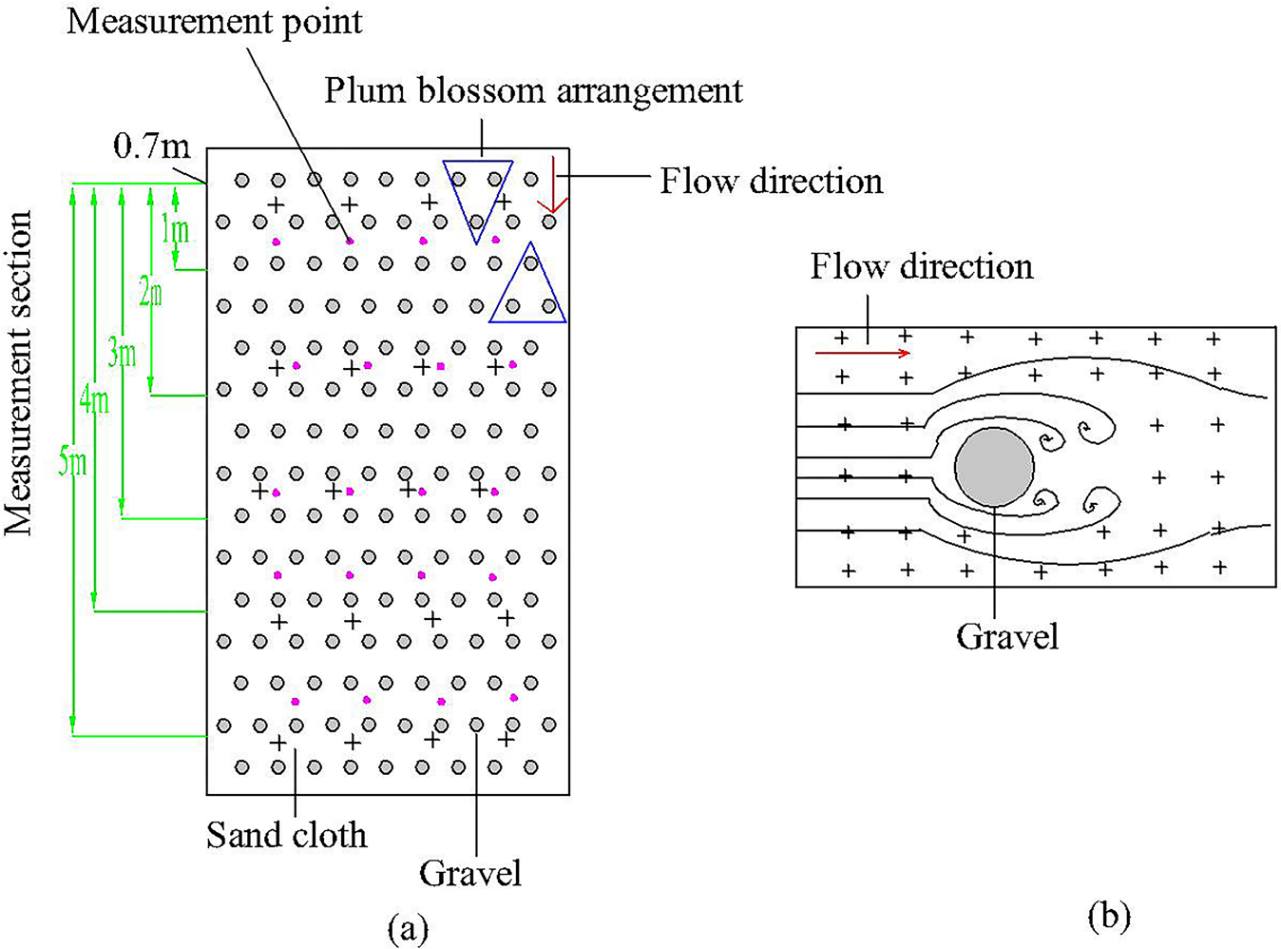
Schematic diagrams of the (**a**) measurement sections and measurement points and (**b**) flow phenomena around gravel. Note: The five green lines refer to five measurement sections; twenty purple-red points are twenty measurement points; two blue triangles refer to the arrangement of gravels in the form of plum blossoms; two bright-red arrows indicate the direction of water flow; “+” symbols refer to sand cloth; and grey-filled circles refer to gravel.

For each test, five measurement sections were set along the slope direction (Fig. 3). The initial measurement section was 0.7 m at the inlet of the hydraulic flume. The distance between each measurement section was 1 m. The five measurement sections were 1.7, 2.7, 3.7, 4.7 and 5.7 m, respectively. Four measurement points in each measurement section were set at 0.1 m intervals. Accordingly, there were 20 measurement points on the slopes. Because of the flow phenomenon around gravel, measurement sections and measuring points on gravel-covered slopes were located between the two plastic hemispheres. In addition, the backwater area and the wake area were avoided to eliminate the influence of water surface inhomogeneity on the test results as much as possible.

Flow depth was measured using a digital level probe (SX40-A, Chongqing Hydrological Equipment Factory) with a precision of 0.01 mm. The flow depth of each point was measured with three replicates. The average of 60 flow depths was considered the flow depth for that combination. Water temperature was measured using a mercury thermometer with an accuracy of 0.1 °C at the beginning and end of the test to calculate the kinematic viscosity coefficient of the water flow.

### Data analysis

The average flow velocity (*u*) can be calculated by the mean flow depth. *u* is calculated by Eq. (1)^32^:

1 $$u=Q/h{b}_{e}$$

where *u* is the mean flow velocity (m/s), *Q* is the flow discharge (m^3^/s), and *h* is the mean flow depth (m). The effect of gravel on the unit flow discharge was considered. The effective runoff width was not equal to the hydraulic flume width because of the protuberant obstacles, and it can be calculated using Eq. (2)^33^:

2 $${b}_{e}=(1-Cr)b$$

where *b*_*e*_ is the effective runoff width (m), *b* is the hydraulic flume width (m), and *Cr* is gravel coverage degree (%). *q* is then computed by dividing the flow discharge *Q* by *b*_*e*_.

The Reynolds number (*Re*) represented the flow regime condition by the ratio of the runoff inertial force to the viscous force. *Re* is calculated by Eq. (3)^34^:

3 $$Re=\frac{uh}{{v}_{m}}=\frac{q}{{v}_{m}}$$

where *h* is the mean flow depth (m) and *v*_*m*_ is the viscosity coefficient of water flow (cm^2^/s), which can be calculated using Eq. (4)^34^:

4 $${v}_{m}=0.01775/(1+0.0337t+0.000221{t}^{2})$$

where *t* is the water flow temperature (°C).

The Froude number (*Fr*) was defined as the ratio of inertia forces to gravitational forces, which reflects the interaction between the flow depth and flow velocity. *Fr* is calculated by Eq. (5)^35^:

5 $$Fr=\frac{u}{\sqrt{gh}}$$

where *g* is gravitational acceleration (m/s^2^).

The resistance coefficient (*f*) reflected the resistance of the underlying surface to overland flow and is calculated by Eq. (6)^35^:

6 $$f=\frac{8gRJ}{{u}^{2}}$$

where *J* is the hydraulic gradient.

The Nash Sutcliffe efficiency coefficient (*NES*) was used to test the simulation effect of the hydraulic model and is calculated by Eq. (7)^36^:

7 $$NSE=1-\frac{{\sum }_{i=1}^{n}{({O}_{i}-{P}_{i})}^{2}}{{\sum }_{i=1}^{n}{({O}_{i}-\bar{O})}^{2}}$$

where *O*_*i*_ is the measured value, *P*_*i*_ is the analogue value, $\bar{O}$ is the average value of the measured values, and *n* is the sample number. Values of *NSE* closer to 1 indicate a better simulation effect of the model.

### Statistical analysis

All statistical analyses were performed in the SPSS v.19.0 environment (SPSS Inc., Chicago, Illinois, United States). A correlation matrix of the Pearson correlation coefficient was used to analyze the correlations between the hydraulic parameters and gravel coverage degrees, slope gradients and flow discharges. A regression analysis was implemented to quantify the relationship between independent and dependent variables.

## Results and Discussion

### Flow velocity

The mean flow velocity (u) ranged from 0.12 to 0.68 m/s on slopes without gravel and from 0.06 to 0.63 m/s on slopes with gravel. Gravel reduced u by 1.20–58.95% in comparison to the slopes without gravel. Under various experimental conditions, gravel had the largest impact on u when the gravel coverage degree was 30%, the slope gradient was 2° and the flow discharge was 22.52 L/min, and the rate of reduction was 58.95%. Gravel had the lowest impact on u when the gravel coverage degree was 10%, the slope gradient was 10° and the flow discharge was 22.52 L/min, and the rate of reduction was 1.20%.

**Figure 4:**
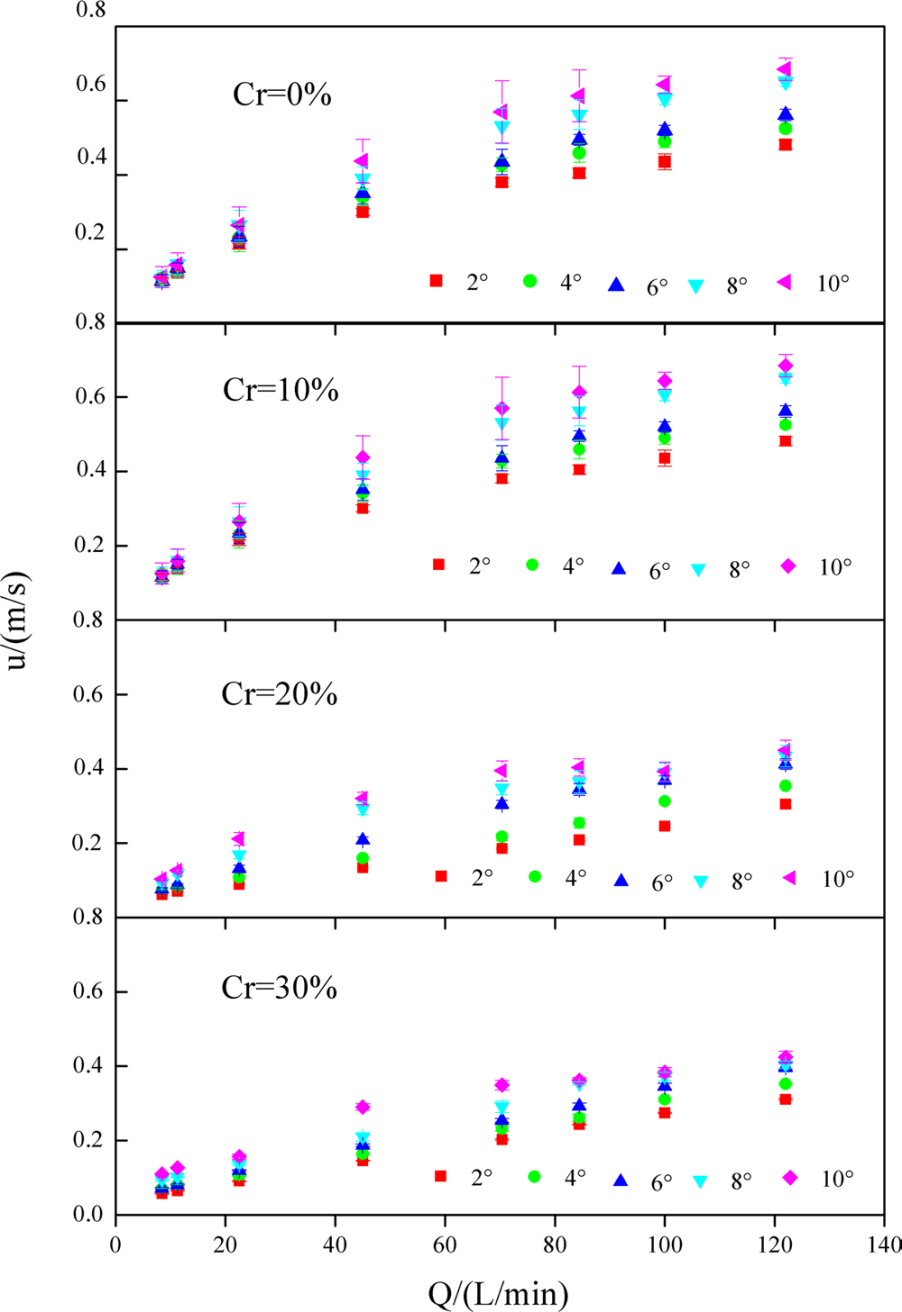
Mean flow velocity (*u*) under various experimental conditions.

*u* increased significantly with flow discharge, and the slope gradient increased and the gravel coverage degree decreased (Fig. 4). On slopes with a gravel coverage degree of 0%, when the flow discharge increased from 8.44 to 122 L/min, *u* increased from 0.11 to 0.48 m/s, 0.12 to 0.52 m/s, 0.12 to 0.56 m/s, 0.12 to 0.65 m/s, and 0.13 to 0.68 m/s for slope gradients of 2°, 4°, 6°, 8°, and 10°, respectively. On slopes with a gravel coverage degree of 10%, when the flow discharge increased from 8.44 to 122 L/min, *u* increased from 0.07 to 0.32 m/s, 0.09 to 0.38 m/s, 0.09 to 0.48 m/s, 0.09 to 0.58 m/s, and 0.10 to 0.63 m/s for slope gradients of 2°, 4°, 6°, 8°, and 10°, respectively. On slopes with a gravel coverage degree of 20%, when the flow discharge increased from 8.44 to 122 L/min, *u* increased from 0.06 to 0.30 m/s, 0.07 to 0.35 m/s, 0.08 to 0.41 m/s, 0.09 to 0.43 m/s, and 0.10 to 0.45 m/s for slope gradients of 2°, 4°, 6°, 8°, and 10°, respectively. On slopes with a gravel coverage degree of 30%, when the flow discharge increased from 8.44 to 122 L/min, *u* increased from 0.06 to 0.29 m/s, 0.06 to 0.34 m/s, 0.07 to 0.40 m/s, 0.09 to 0.40 m/s, and 0.11 to 0.42 m/s for slope gradients of 2°, 4°, 6°, 8°, and 10°, respectively.

*u* was significantly positively correlated with *Q* (r = 0.716, P < 0.01**) and *J* (r = 0.674, P < 0.01**) and significantly negatively correlated with *Cr* (r = −0.846, P < 0.01**) (Table 1). The relationship between *u* and *Q* and *J* and *Cr* presented a power function (Table 2). The *NSE* of Equation (10) was 0.962, demonstrating that the equation was superior. According to Equation (10), the exponent of *Cr* (1.539) was higher than that of *Q* (0.574) and *J* (0.340), which indicated that *u* was mainly affected by *Cr* followed by *Q* and *J*.

**Table 1:** Correlation analysis for hydraulic parameters and flow discharges, slope gradients and gravel coverage degrees.

	*u*	*h*	*Re*	*Fr*	*f*
Q	0.716**	0.729**	0.989**	0.623**	−0.868**
J	0.674**	−0.678**	−0.008	0.699**	−0.038
Cr	−0.846**	0.774**	0.712**	−0.851**	0.962**

Notes: N = 160, *Cr* = gravel coverage degree, *Q* = flow discharge, *J* = slope gradient, *u* = average velocity, *h* = mean depth, *Re* = Reynolds number, *Fr* = Froude number, and *f* = Darcy-Weisbach resistance coefficient.

*P < 0.05 **P < 0.01.

**Table 2:** Function relations between the average velocity (*u*) and gravel coverage degree (*Cr*) and flow discharge (*Q*) and slope gradient(*J*) under different conditions.

*Cr*	Equation	*R*^2^	*NSE*	*n*	
*Cr* = 0%	*u* = 0.076*Q*^0.551^ sin *J*^0.240^	0.981	0.981	40	(8)
*Cr* ≠ 0%	$\,u=0.082{(1-Cr)}^{1.165}{Q}^{0.588}\,\sin \,{J}^{0.406}$	0.966	0.966	120	(9)
All slopes	$u=0.083{(1-Cr)}^{1.539}{Q}^{0.574}\,\sin \,{J}^{0.340}$	0.962	0.962	160	(10)

*u* is not only an index to describe hydrological processes under different erosion conditions but also the basis for calculating other hydraulic variables, such as the flow shear force, flow power and unit flow power, which are used to simulate soil separation and sediment transport^37,38^. A larger flow velocity indirectly increased the runoff power, resulting in a larger capacity for sediment transportation^39,40^. In our study, gravel had the effect of retarding *u* compared with the slopes without gravel cover. The reasons were as follows. On the one hand, gravel prolonged the flow path and increased losses along the way. On the other hand, the eddies that formed around the gravel increased the local losses^41,42^. Both of them greatly decreased the kinetic energy of water flow. Consequently, *u* decreased.

With the increase in gravel coverage degree, the reduction effect of gravel enhanced *u*. This finding was consistent with studies from Foster *et al*.^43^. As the flow discharge and the slope gradient increased, *u* increased. The relationship between *u* and flow discharge and slope gradient was a power function and showed a positive correlation. Nearing *et al*.^44^ and Foster *et al*.^45^ reported similar results. However, Zhang *et al*.^46^ thought that *u* of overland flow was mainly controlled by the flow discharge, and the slope gradient had little effect on the *u* on bare slopes through indoor drainage and scouring experiment. King and Norton^47^ and Ali *et al*.^37^ considered that the slope gradient had no significant effect on *u* for mobile beds covered with soil. The reason for this difference may be related to the different underlying surface conditions.

### Flow depth

The mean flow depth (*h*) ranged from 2.24 to 8.45 mm on slopes without gravel and from 3.03 to 18.69 mm on slopes with gravel. Gravel increased *h* by 0.12–2.41 times in comparison to the slopes without gravel. Under various experimental conditions, gravel had the largest impact on *h* when the gravel coverage degree was 30%, the slope gradient was 2°, and the flow discharge was 22.52 L/min; and the increased time was 2.41. Gravel had the lowest impact on *h* when the gravel coverage degree was 10%, the slope gradient was 10° and the flow discharge was 22.52 L/min; and the increased time was 0.12.

**Figure 5:**
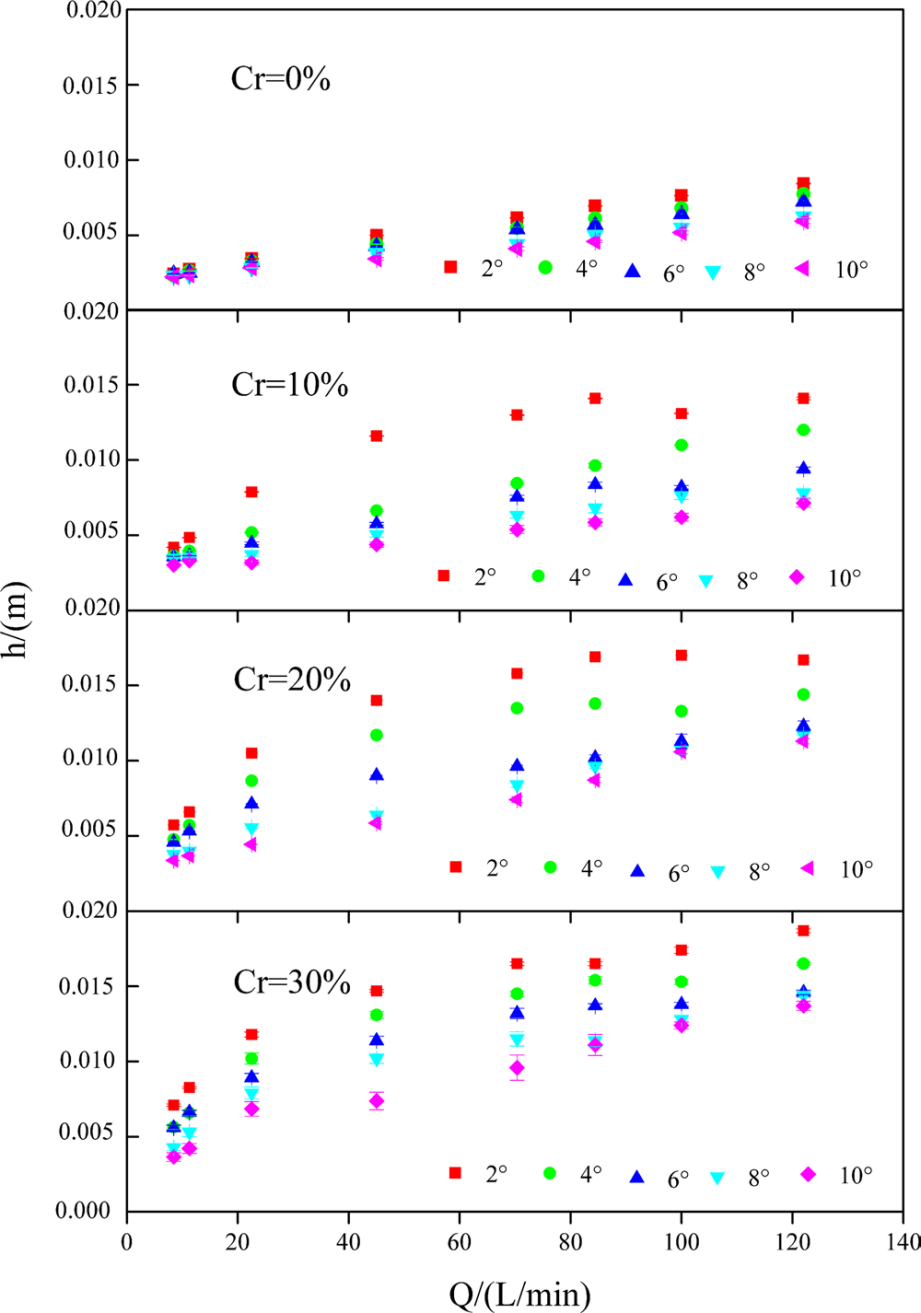
Mean flow depth (*h*) under various experimental conditions.

*h* increased significantly with flow discharge, the gravel coverage degree increased and the slope gradient decreased (Fig. 5). On slopes with a gravel coverage degree of 0%, when the flow discharge increased from 8.44 to 122 L/min, *h* increased from 2.44 to 8.45 mm, 2.32 to 7.75 mm, 2.44 to 7.25 mm, 2.32 to 6.25 mm, and 2.24 to 5.94 mm for slope gradients of 2°, 4°, 6°, 8°, and 10°, respectively. On slopes with a gravel coverage degree of 10%, when the flow discharge increased from 8.44 to 122 L/min, *h* increased from 4.20 to 14.10 mm, 3.65 to 11.97 mm, 3.56 to 9.41 mm, 3.49 to 7.80 mm, and 3.03 to 7.14 mm for slope gradients of 2°, 4°, 6°, 8°, and 10°, respectively. On slopes with a gravel coverage degree of 20%, when the flow discharge increased from 8.44 to 122 L/min, *h* increased from 5.72 to 16.68 mm, 4.77 to 14.35 mm, 4.59 to 12.31 mm, 3.77 to 11.69 mm, and 3.38 to 11.29 mm for slope gradients of 2°, 4°, 6°, 8°, and 10°, respectively. On slopes with a gravel coverage degree of 30%, when the flow discharge increased from 8.44 to 122 L/min, *h* increased from 7.10 to 18.69 mm, 5.64 to 16.49 mm, 5.59 to 14.59 mm, 4.28 to 14.40 mm, and 3.65 to 13.69 mm for slope gradients of 2°, 4°, 6°, 8°, and 10°, respectively.

*h* was significantly positively correlated with *Q* (r = 0.729, P < 0.01**) and *Cr* (r = 0.774, P < 0.01**) and significantly negatively correlated with *J* (r = −0.678, P < 0.01**) (Table 1). The relationship between *h* and *Q* and *J* and *Cr* presented a power function (Table 3). The *NSE* of Equation (13) was 0.922, demonstrating that the equation was superior. According to the Equation (13), the exponent of *Cr* (2.472) was higher than that of *Q* (0.402) and *J* (0.333), which indicated that *h* was mainly affected by *Cr* followed by *Q* and *J*.

**Table 3:** Function relations between the mean depth (*h*) and gravel coverage degree (*Cr*) and flow discharge (*Q*) and slope gradient (*J*) under different conditions.

*Cr*	Equation	*R*^2^	*NSE*	*n*	
*Cr* = 0%	$h=0.00060{Q}^{0.408}\,\sin \,{J}^{-0.181}$	0.984	0.965	40	(11)
*Cr* ≠ 0%	$h=0.00048{(1-Cr)}^{-1.941}{Q}^{0.399}\,\sin \,{J}^{-0.383}$	0.978	0.930	120	(12)
All slopes	$h=0.00046{(1-Cr)}^{-2.472}{Q}^{0.402}\,\sin \,{J}^{-0.333}$	0.974	0.922	160	(13)

*h* affects the transport process of sediment particles and the extent of soil erosion^48^. Nevertheless, the water depth of overland flow is very shallow, which is strongly affected by the external disturbance such as surface coverage. Meanwhile, limited by the measurement methods, it is not easy to directly measure the water depth of overland flow in the field. Therefore, the study on the relationships between the water depth and flow discharge, slope gradient and coverage degree have not yet in-depth research at present^49^. In this experiment, the roughness is stable at the bottom of the flume, there is no problem of sediment deposition in flowing water, so the water depth can be measured accurately. Gravel had the function of backup *h* in this research. This was mainly attributed to the reduction in *b*_*e*_ and *u* on gravel-covered slopes. *h* increased depending on Eq. (1). A larger flow depth improved the runoff shear stress, leading to greater disturbance to the soil surface^50^.

With the increase in gravel coverage degree, the backup effect of gravel on *h* was more obvious. Bunte^41^ presented similar results. On the contrary, Fu *et al*.^51^ obtained that *h* tended to decrease with the increase of gravel coverage degree through artificial rainfall simulation experiment. This discrepancy may be related to soil infiltration. In addition, under an identical gravel coverage degree, *h* increased with increasing flow discharge and slope gradient. Similar research results were obtained by Zhang *et al*.^46^.

### Reynolds number

The Reynolds number (*Re*) is the ratio of inertial forces to the viscous force, and it represents the overland flow regime conditions. As the Reynolds number increases, the probability of a turbulent overland flow also increases. Hydraulic theory states that overland flow is turbulent when the *Re* is greater than 6500 and laminar when the *Re* is less than 580. Overland flow is transitional when the *Re* is between 580 and 6500^52^. Overland flow is laminar when the flow discharge is less than 22.52 L/min. Conversely, overland flow is transitional.

Gravel increased the *Re* by 2.94–33.03% in comparison with the slopes without gravel. A higher *Re* may be due to the presence of gravel reducing the *b*_*e*_. Among all the treatments, gravel had the largest impact on the *Re* when the gravel coverage degree was 30%, the slope gradient was 2° and the flow discharge was 122 L/min, and the rate of increase was 33.03%. Gravel had the least impact on the *Re* when the gravel coverage degree was 10%, the slope gradient was 10° and the flow discharge was 8.44 L/min, and the rate of increase was 2.94%.

**Figure 6:**
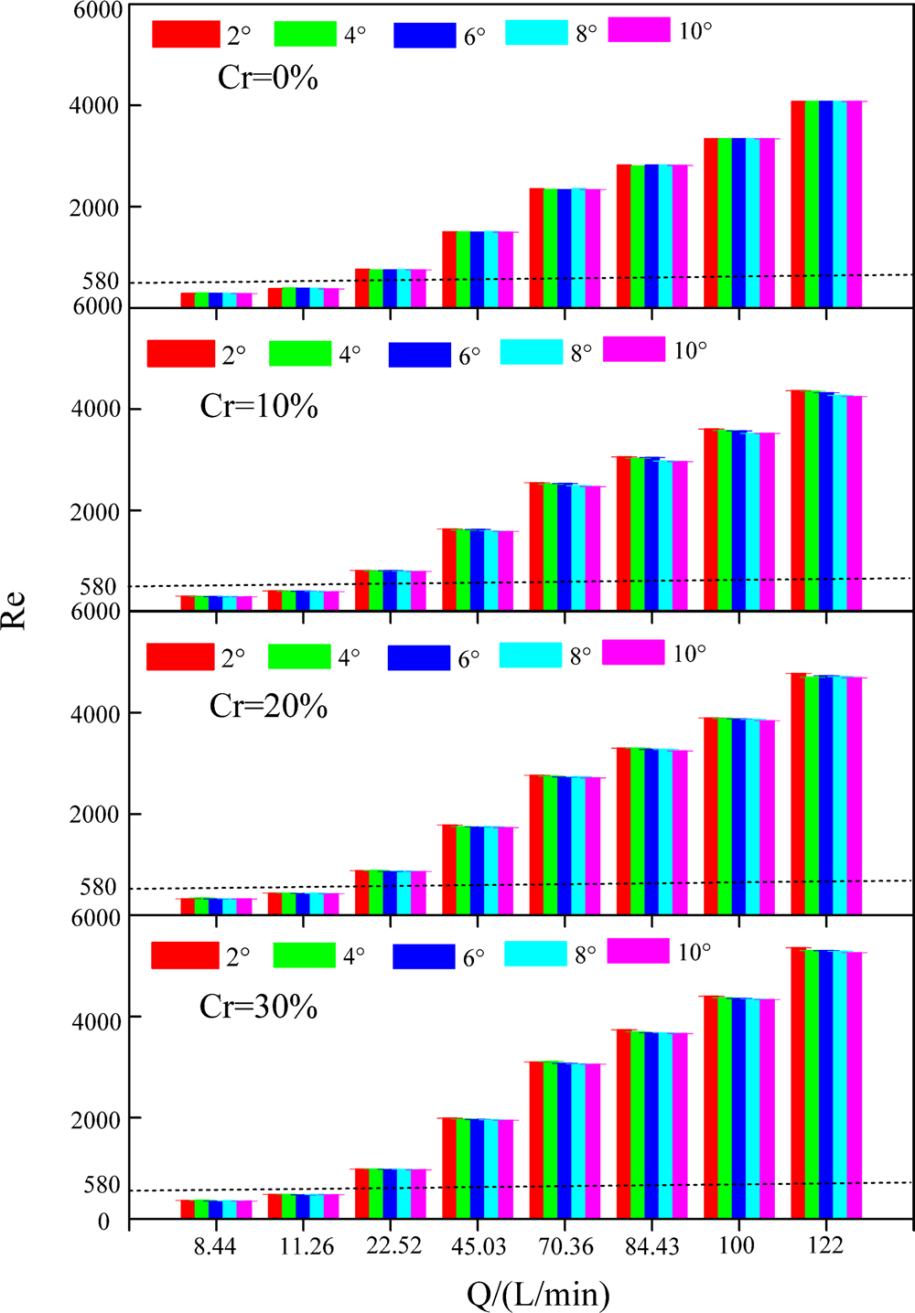
Reynolds number (*Re*) under various experimental conditions.

The *Re* did not change significantly with increasing slope gradient^53^ (Fig. 6). However, the present result was inconsistent with the findings of Zhai *et al*.^54^. This difference may be caused by the water flow temperature. The *Re* was positively correlated with the unit width discharge and negatively correlated with the viscosity coefficient (see Eq. (3)). Under an identical flow discharge and identical gravel coverage degree, the unit width discharge had little difference under different slope gradients. Thus, the *Re* was mainly affected by the viscosity coefficient under gravel covered slopes with different slope gradients, and this coefficient was primarily affected by the water flow temperature. The experiment took place in autumn, and the water flow temperature changed little with the weather. Therefore, the viscosity coefficient of water flow fluctuated slightly under gravel covered slopes with different slope gradients, which led to the similar *Re* values under the five slope gradients.

The *Re* increased significantly with increasing flow discharge and gravel coverage degree (Fig. 6). On slopes with a gravel coverage degree of 0%, when the flow discharge increased from 8.44 to 122 L/min, the *Re* increased from 281 to 4077, which represented an increase of 13.49 times. On slopes with a gravel coverage degree of 10% under identical conditions, the *Re* increased from 296 to 4368, which represented an increase of 13.78 times. On slopes with a gravel coverage degree of 20% under identical conditions, the *Re* increased from 320 to 4770, which represented an increase of 13.90 times. On slopes with a gravel coverage degree of 30% under identical conditions, the *Re* increased from 356 to 5357, which represented an increase of 14.06 times.

The *Re* was significantly positively correlated with *Q* (r = 0.989, P < 0.01**) and *Cr* (r = 0.712, P < 0.01**) and not correlated with *J* (r = −0.008, P > 0.01) (Table 1). The relationship among *Re*, *Q* and *Cr* presented a power function (Table 4). The *NSE* of Equation (16) was 0.999, demonstrating that the equation was superior. According to Equation (16), the exponent of *Q* (0.995) was higher than that of *Cr* (0.760), which showed that the *Re* was mainly affected by *Q* followed by *Cr*.

**Table 4:** Function relations between the Reynolds number (*Re*) and gravel coverage degree (*Cr*) and flow discharge(*Q*) under different conditions.

*Cr*	Equation	*R*^2^	*NSE*	*n*	
*Cr* = 0%	$Re=33.510{Q}^{0.999}$	0.992	0.999	40	(14)
*Cr* ≠ 0%	$Re=33.345{(1-Cr)}^{-0.819}{Q}^{0.994}$	0.954	0.999	120	(15)
All slopes	$Re=33.766{(1-Cr)}^{-0.760}{Q}^{0.995}$	0.995	0.999	160	(16)

The *Re* is an important parameter for measuring the soil disturbance caused by overland flow. The *Re* increased with increasing the flow discharge and the gravel coverage degree^41^. Our results differed from those presented by Li *et al*.^55^, who found that the *Re* decreased with increases in the gravel coverage degree when the gravel coverage degree and flow discharge were relatively small. As the gravel coverage degree and the flow discharge increased, the trend gradually diminished and then reversed. Furthermore, Salman *et al*.^56^ and Rieke-Zapp *et al*.^12^ suggested that the *Re* did not change significantly with increases in the gravel coverage degree based on their experimental data. The discrepancy could be related to a greater amount of surface roughness and a higher infiltration rate on natural gravel-covered slopes.

### Froude number

The *Fr* reflects the ratio of inertial force to gravity and characterizes the overland flow pattern conditions. Inertial force plays a leading role in water flow, and supercritical flow occurs when *Fr* < 1. Gravity is equal to inertial force in water flow, and critical flow occurs when *Fr* = 1.

The *Fr* varied from 0.75 to 2.89 on slopes without gravel and from 0.21 to 2.42 on slopes with gravel. Gravel reduced the *Fr* by 6.83–77.31% in comparison to the slopes without gravel. Under various experimental conditions, gravel had the largest impact on the *Fr* when the gravel coverage degree was 30%, the slope gradient was 2° and the flow discharge was 22.52 L/min, and the rate of reduction was 77.31%. Gravel had the least impact on the *Fr* when the gravel coverage degree was 10%, the slope gradient was 10° and the flow discharge was 22.52 L/min, and the rate of reduction was 6.83%.

**Figure 7:**
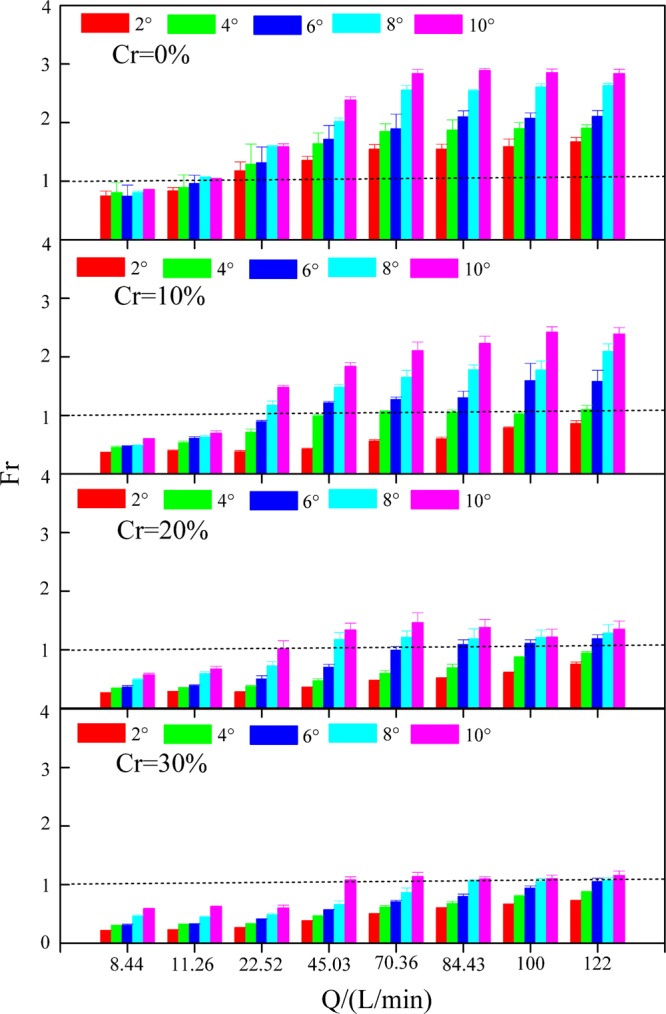
Froude number (*Fr*) under various experimental conditions.

Under identical gravel coverage degree conditions, the *Fr* increased significantly with increasing flow discharge and slope gradient. The flow pattern developed from a subcritical flow to a supercritical flow. However, with the increase in gravel coverage degree, the *Fr* tended to decrease. The flow pattern developed from supercritical flow to subcritical flow (Fig. 7). On slopes with a slope gradient of 2°, when the flow discharge increased from 8.44 to 122 L/min, the *Fr* increased from 0.74 to 1.67, 0.36 to 0.86, 0.25 to 0.75, and 0.21 to 0.72 when the gravel coverage degree was 0%, 10%, 20% and 30%, respectively. On slopes with a slope gradient of 4°, when the flow discharge increased from 8.44 to 122 L/min, the *Fr* increased from 0.80 to 1.90, 0.45 to 1.10, 0.34 to 0.94, and 0.30 to 0.87 when the gravel coverage degree was 0%, 10%, 20% and 30%, respectively. On slopes with a slope gradient of 6°, when the flow discharge increased from 8.44 to 122 L/min, the *Fr* increased from 0.74 to 2.10, 0.47 to 1.58, 0.36 to 1.18, and 0.30 to 1.05 when the gravel coverage degree was 0%, 10%, 20% and 30%, respectively. On slopes with a slope gradient of 8°, when the flow discharge increased from 8.44 to 122 L/min, the *Fr* increased from 0.80 to 2.63, 0.48 to 2.09, 0.48 to 1.28, and 0.45 to 1.07 when the gravel coverage degree was 0%, 10%, 20% and 30%, respectively. On slopes with a slope gradient of 10°, when the flow discharge increased from 8.44 to 122 L/min, the *Fr* increased from 0.84 to 2.83, 0.59 to 2.39, 0.57 to 1.35, and 0.58 to 1.15 when the gravel coverage degree was 0%, 10%, 20% and 30%, respectively.

The *Fr* was significantly positively correlated with *Q* (r = 0.623, P < 0.01**) and *J* (r = 0.699, P < 0.01**) and significantly negatively correlated with *Cr* (r = −0.851, P < 0.01**) (Table 1). The relation between *Fr* and *Q* and *J* and *Cr* presented a power function (Table 5). The *NSE* of Equation (19) was 0.931, demonstrating that the equation was superior. According to Equation (19), the exponent of *Cr* (2.936) was higher than that of *Q* (0.378) and *J* (0.498), which indicated that the *Fr* was mainly affected by *Cr* followed by *J* and *Q*.

**Table 5:** Function relations between the Froude number (*Fr*) and gravel coverage degree (*Cr*) and flow discharge (*Q*) and slope gradient (*J*) under different conditions.

*Cr*	Equation	*R*^2^	*NSE*	*n*	
*Cr* = 0%	$Fr=0.978{Q}^{0.362}\,\sin \,{J}^{0.356}$	0.928	0.928	40	(17)
*Cr* ≠ 0%	$\,Fr=1.480{(1-Cr)}^{2.456}{Q}^{0.390}\,\sin \,{J}^{0.664}$	0.931	0.931	120	(18)
All slopes	$\,Fr=1.221{(1-Cr)}^{2.936}{Q}^{0.378}\,\sin \,{J}^{0.498}$	0.931	0.931	160	(19)

The flow depth and the flow velocity determine the sediment carrying capacity and runoff shear force. *Fr* reflects the relationship between the flow depth and the flow velocity. Gravel retarded the *Fr*_,_ which indicated that the presence of gravel reduced the turbulence of water flow. This effect was primarily attributed to the reduction in flow velocity and increase in flow depth due to gravel cover. Accordingly, the *Fr* decreased depending on expression (5). Salman *et al*.^56^ and Guo *et al*.^42^ showed that the *Fr* was mainly controlled by the underlying surface and tended to decrease as the gravel coverage degree increased and the flow pattern transformed from supercritical flow to subcritical flow, which was consistent with the results of this study. In addition, as the flow discharge and the slope gradient increased, the *Fr* increased. This finding was consistent with studies from Jing *et al*.^57^ and Ye *et al*.^58^. The larger *Fr* meant smaller runoff shear force and a larger sediment carrying capacity^38^.

### Resistance coefficient

The Darcy-Weisbach resistance coefficient (*f*) varied from 0.10 to 1.94 on slopes without gravel and from 0.24 to 8.88 on slopes with gravel. Gravel increased *f* by 0.15–18.42 times in comparison to the slopes without gravel. Under various experimental conditions, gravel had the largest impact on *f* when the gravel coverage degree was 30%, the slope gradient was 2°, and the flow discharge was 22.52 L/min, thus presenting an increase of 18.42 times. Gravel had the least impact on *f* when the gravel coverage degree was 10%, the slope gradient was 10° and the flow discharge was 22.52 L/min, thus presenting an increase of 0.15 times.

**Figure 8:**
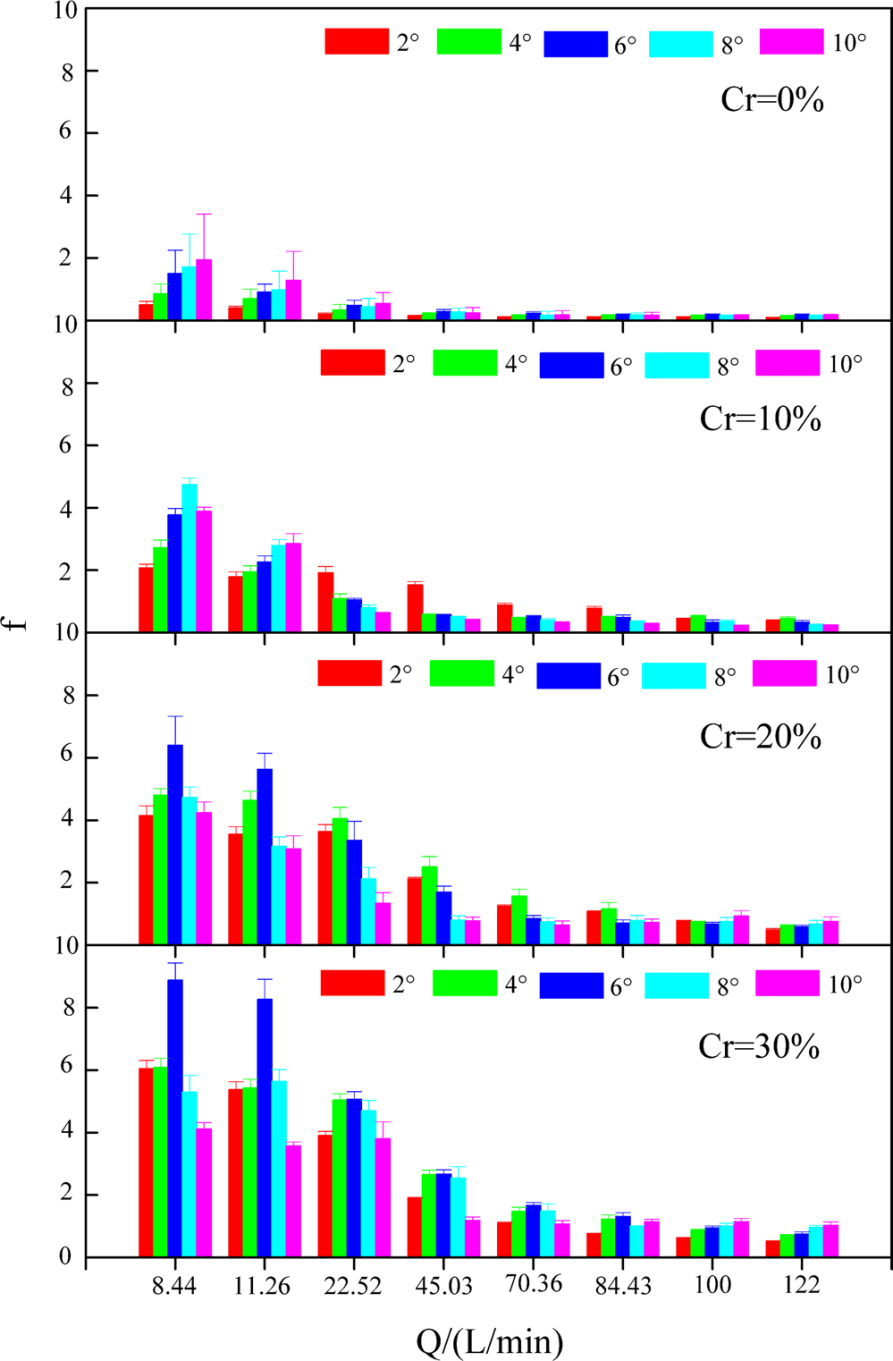
Resistance coefficient (*f*) under various experimental conditions.

*f* did not demonstrate significant regularity with increasing slope gradient^38^ (Fig. 8). However, there were some inconsistent opinions on the relationship between *f* and slope gradient. Yao^59^ considered that *f* was positively correlated with slope gradient. Pan and Shangguan^60^ thought that *f* was negatively correlated with slope gradient. The reason for the difference may be explained by the different experimental conditions and different experimental methods.

*f* decreased significantly with increasing flow discharge and decreasing gravel coverage (Fig. 8). On slopes with a gravel coverage degree of 0%, when the flow discharge increased from 8.44 to 122 L/min, *f* decreased from 1.93 to 0.10, which represented a reduction of 1.83. On slopes with a gravel coverage degree of 10% under identical conditions, *f* decreased from 4.73 to 0.23, which represented a reduction of 4.50. On slopes with a gravel coverage degree of 20% under identical conditions, *f* decreased from 6.39 to 0.49, which represented a reduction of 5.90. On slopes with a gravel coverage degree of 30% under identical conditions, *f* was decreased from 8.88 to 0.52, which represented a reduction of 8.36.

*f* was significantly positively correlated with *Cr* (r = 0.962, P < 0.01**) and significantly negatively correlated with *Q* (r = −0.868, P < 0.01**) but not correlated with *J* (r = −0.038, P > 0.01) (Table 1). The relation between *f* and *Q* and *Cr* presented a power function (Table 6). The *NSE* of Equation (22) was 0.841, demonstrating that the equation was superior. According to Equation (22), the exponent of *cr* (3.652) was higher than that of *Q* (0.701), which indicated that *f* was mainly affected by *Cr* followed by *Q*.

**Table 6:** Function relations between the Darcy-Weisbach resistance coefficient (*f*) and gravel coverage degree (*Cr*) and flow discharge (*Q*).

*Cr*	Equation	*R*^2^	*NSE*	*n*	
*Cr* = 0%	$f=10.540{Q}^{-1.003}$	0.748	0.748	40	(20)
*Cr* ≠ 0%	$\,f=10.638{(1-Cr)}^{-2.929}{Q}^{-0.699}$	0.840	0.840	120	(21)
All slopes	$\,f=8.461{(1-Cr)}^{-3.652}{Q}^{-0.701}$	0.841	0.841	160	(22)

*f* is an important variable in the soil erosion model and reflects the resistance of the underlying surface to overland flow. With the increase in the gravel coverage degree, the contact area between gravel and water flow increased, and flow resistance played a dominant role, leading to an increase in overland flow resistance. A higher *f* on gravel-covered slopes indicated that overland flow was not only affected by particle resistance but also by the flow resistance around gravel. Engman^61^ studied the resistance coefficient of farmland based on data collected from runoff plots. Savat^62^ described the resistance coefficients for various surfaces. Gilley *et al*.^63^ constructed a resistance coefficient equation under pebble cover based on a flume experiment. In these studies, *f* decreased with increasing flow discharge and decreasing gravel coverage degree, and these findings are consistent with the results of this study.

## Conclusions

Gravel can change water flow patterns, reduce flow velocity, and increase water depth and resistance. The *h*, *Re*, and *f* values on gravel-covered slopes were 0.12–2.41 times, 2.94–33.03%, and 0.15–18.42 times higher, respectively, than those of the slopes without gravel. However, the *u* and *Fr* values on gravel-covered slopes were 1.20–58.95% and 6.83–77.31% lower, respectively, than those of the slopes without gravel.

*Q*, *J* and *Cr* affected the hydraulic characteristics of overland flow. *u* and *Fr* were positively correlated with both *Q* and *J* and negatively correlated with *Cr*. *h* was positively correlated with both *Q* and *Cr* and negatively correlated with *J*. The *Re* and *f* were less affected by *J* and positively correlated with *Cr*. However, the *Re* increased and *f* decreased with an increase in *Q*.

The relationships among hydraulic parameters and *Q*, *J* and *Cr* were described by good power functions (*R*^2^ > 0.7, *NSE* > 0.7). The *Re* was most affected by *Q*, and the other hydraulic parameters were most affected by *Cr*. The results provide insights into the mechanism underlying gravel’s control of soil erosion and can guide the ecological construction of soil and water conservation. Further research is needed on the effects of different particle sizes and arrangement patterns on the hydraulic characteristics of gravel-covered slopes.
